# Efficacy and influencing factors of immunosuppressive therapy combined with or without eltrombopag in children with severe aplastic anemia

**DOI:** 10.3389/fmed.2025.1688771

**Published:** 2025-11-21

**Authors:** Jingchen Gao, Hui Yang, Meiling Liao, Mingzhu Luo, Ye Feng, Jiebin Qin, Xianmin Guan, Xianhao Wen

**Affiliations:** Department of Hematology and Oncology, Children’s Hospital of Chongqing Medical University, National Clinical Research Center for Child Health and Disorders, Ministry of Education Key Laboratory of Child Development and Disorders, Chongqing Key Laboratory of Child Rare Diseases in Infection and Immunity, Chongqing, China

**Keywords:** aplastic anemia, children, eltrombopag, efficacy, influencing factors

## Abstract

**Background:**

To compare the efficacy of eltrombopag (EPAG) combined with standard immunosuppressive therapy (IST), EPAG with cyclosporine or cyclosporine alone in children with severe aplastic anemia (SAA).

**Methods:**

This is a retrospective study. The patients were categorized as three groups: Group A (EPAG + rabbit antithymocyte globulin + cyclosporine, *n* = 12), Group B (EPAG + cyclosporine, *n* = 13), and Group C (cyclosporine alone, *n* = 16). The overall remission rate (ORR) of each group at 1, 3, 6, and 12 months of treatment was evaluated.

**Results:**

There was no significant difference in the ORR among Groups A, B, and C at 1, 3, 6 and 12 months (*P* > 0.05). The incidence rates of adverse reactions in each group at 6 months were 36.4% (4/11), 58.3% (7/12), and 69.2% (9/13), respectively (*P* = 0.264). Patients with a duration from diagnosis to receiving EPAG treatment of ≤ 60 days, a diagnosis of SAA, a platelet (PLT) count ≥ 15 × 10^9^/L, a white blood cell (WBC) count of ≥ 2.0 × 10^9^/L, N% of ≥ 40%, a lymphocyte count of ≥ 1.0 × 10^9^/L, and a CD4^+^/CD8^+^ ratio of ≥ 1.5 were more likely to achieve a hematopoietic response. No clonal evolution was observed in any of the patients.

**Conclusion:**

EPAG combined with IST shows comparable efficacy to cyclosporine alone in children with SAA, along with a favorable safety profile. Patients with earlier initiation of EPAG and preserved residual hematopoietic function are more likely to achieve remission, without serious adverse reactions or significant clonal evolution.

## 1 Introduction

Aplastic anemia (AA) is a serious blood disease that typically occurs in childhood and is characterized by pancytopenia in peripheral blood and bone marrow failure syndrome resulting from various known or unknown etiologies. Its annual incidence rate is 0.74/100,000 people in China. In children, AA is predominantly acquired (1). In accordance with the relevant diagnostic criteria of the Diagnosis and Treatment Guidelines for Pediatric Aplastic Anemia (2019 Edition) (2), AA can be divided into severe AA (SAA), very severe AA (VSAA), and non-severe AA (NSAA), in which SAA and VSAA are difficult to treat and have high mortality rates. Hematopoietic stem cell transplantation (HSCT) offers a curative approach for AA, characterized by rapid efficacy, durable remission, and low rates of long-term relapse or clonal evolution. Nevertheless, its feasibility is limited by donor availability, HLA compatibility, and transplant timing. Consequently, immunosuppressive therapy (IST) serves as the standard treatment for patients without an appropriate donor. Standard IST consisting of antithymocyte globulin (ATG) combined with Cyclosporine A (CsA). Currently, rabbit antithymocyte globulin (rATG) and horse antithymocyte globulin (hATG) are the two most commonly used forms of ATG. The initial response rate of the basic IST regimen (hATG+CsA) is approximately 60–65% (3). Approximately 2/3 of patients respond to IST, and 1/3 of patients are refractory to IST. This may be related to the low number and abnormal quality of residual hematopoietic stem cells and progenitor cells (HSPCs) (4).

Eltrombopag (EPAG) is an oral thrombopoietin receptor agonist that is widely used to treat immune thrombocytopenia, SAA, and hepatitis C-associated thrombocytopenia and has demonstrated clinical efficacy (5). EPAG binds to the thrombopoietin receptor and activates the JAK2/STAT5 signaling pathway, thereby promoting megakaryocyte maturation and platelet release (6). Compared with traditional hematopoietic growth factors, EPAG can promote the proliferation and differentiation of hematopoietic stem cells, promote the repair of the bone marrow microenvironment, and improve the hematopoietic function of the bone marrow (7). IST mainly suppresses abnormal immune responses. The combination of the two can stimulate the proliferation and differentiation of bone marrow hematopoietic stem cells and, on the other hand, suppress the inhibitory effect of the immune system on hematopoietic cells. One study reported that the overall remission rate of children with SAA receiving EPAG combined with IST was as high as 50–70%, indicating that these patients had a better hematopoietic response than did those receiving standard IST treatment (8).

However, there is limited data on children in the existing domestic and international studies, and the results of previous studies are also contradictory. This study compared the efficacy of EPAG combined with IST and IST alone in the treatment of children with SAA, analyzed the predictive value of different influencing factors for efficacy, observed the occurrence of adverse reactions and clonal evolution, and monitored the survival rate to provide new insights for accurate and effective treatment of children with SAA.

## 2 Materials and methods

### 2.1 Patients

A total of 80 AA patients who were treated in our hospital between May 2022 and December 2024 were selected. The patients were aged between 0 and 18 years old. Patients who were not followed up after diagnosis and those who underwent HSCT after diagnosis were excluded. A total of 41 patients (29 SAA patients and 12 VSAA patients) were selected for retrospective analysis, and the last follow-up time was February 2025. The inclusion criteria were as follows: (1) met the relevant diagnostic criteria for Diagnosis and Treatment Guidelines for Pediatric Aplastic Anemia (2019 edition) and were confirmed by complete blood count and bone marrow biopsy; and (2) were newly diagnosed patients without an HLA-matched sibling donor and had never received HSCT. The exclusion criteria were congenital bone marrow failure diseases or other acquired bone marrow failure syndromes.

### 2.2 Treatment options

Grouping: Group A: EPAG + rATG + CsA treatment; Group B: EPAG + CsA treatment; Group C: CsA alone treatment. The initial administration of EPAG after diagnosis was not less than 1 month.

Drug administration: ➀ EPAG: initial dose: a. age 6 years and above, 25 mg/time, once a day; b. age 1–5 years, 1.5 mg/kg, once a day. The dosage should be adjusted based on PLT count (maintained ≥ 50 × 10^9^/L). The maximum dose for children aged 6–11 years should not exceed 50 mg/d, and the maximum dose for children aged 12–17 years should not exceed 75 mg/d. ➁ CsA: CsA (5 mg/kg/day) was given orally two times to maintain the plasma concentration at 100–150 ng/mL (trough concentration). ➂ rATG: 2.5 mg/kg/day, intravenous infusion, for five consecutive days. Routine antiallergic drugs were given half an hour before the infusion. ➃ Supportive treatment: When necessary, platelet transfusion, red blood cell suspension, and anti-infective treatment should be given to patients with evidence of infection.

### 2.3 Evaluation of efficacy

Primary endpoint: ORR was evaluated after 1, 3, 6, and 12 months of treatment. The remission criteria were as follows: ➀ Complete remission (CR): absolute neutrophil (ANC) count > 1.5 × 10^9^/L, hemoglobin (Hb) count > 110 g/L, and platelet (PLT) count > 100 × 10^9^/L, independent from red blood cell (RBC) and PLT transfusion. ➁ Partial remission (PR): ANC > 0.5 × 10^9^/L, Hb > 80 g/L, and PLT > 20 × 10^9^/L, independent from both RBC and PLT transfusion. ➂ No remission (NR): The patient did not meet the criteria of CR or PR. Overall remission = complete remission + partial remission (2).

Secondary endpoints: Secondary endpoints included 6–month changes in peripheral blood counts, bone-marrow hematopoiesis, and immune function; the single-lineage response (SLR) rate; overall survival (OS); and clonal evolution. The criteria for a single-lineage response were as follows (9): ➀ Erythrocyte response: Hb increased by ≥ 15 g/L compared with the baseline level (if initially < 90 g/L, the patient did not receive RBC transfusion or the number of RBC units transfused for 8 consecutive weeks was reduced by ≥ 4 units compared with the pretreatment level). ➁ Granulocyte reaction: ANC ≥ 0.5 × 10^9^/L greater than the baseline value (if the initial value was < 0.5 × 10^9^/L, it increased by at least 1-fold compared with the baseline value). ➂ Megakaryocyte reaction: PLT ≥ 20 × 10^9^/L or higher than the baseline value (if the initial value was < 20 × 10^9^/L, it increased by at least 1-fold compared with the baseline value, or there was no PLT transfusion for at least 8 weeks). Clonal evolution was defined as the emergence of a new clone with cytogenetic abnormalities or the transformation of myelodysplastic syndrome (MDS) or acute myeloid leukemia (AML). Event-free survival (EFS) was defined as the time from the start of treatment to the occurrence of any event, including HSCT, death (10).

The influencing factors included age, sex, disease severity, time from diagnosis to receiving EPAG, duration of EPAG use, peripheral blood cell count, erythropoietin levels, thrombopoietin levels, lymphocyte composition, immunoglobulin levels, and bone marrow hematopoietic cell count.

### 2.4 Adverse events

Liver and kidney function, skin rash, gingival hyperplasia, joint and muscle pain, and thromboembolic events were monitored before treatment and at 1,3 and 6 months after treatment. The incidence of adverse reactions in each group during the 6-month follow-up was analyzed.

### 2.5 Statistical methods

Data analysis was performed via SPSS 26.0 software. The continuous variable with a normal distribution were expressed as the means ± standard deviations; the continuous variable with a non-normal distribution were expressed as the medians (ranges); *t*-tests or Mann–Whitney U tests were used for data analysis; the categorical variable were expressed as percentages; and the χ*2* test or Fisher’s exact test was used. Intergroup comparisons were performed using analysis of variance (ANOVA). ROC curves were used to assess the predictive value of relevant indicators for treatment response. Survival curves were drawn via the Kaplan–Meier method, and differences in survival between groups were compared using the log-rank test. Differences with *P* < 0.05 were considered statistically significant.

## 3 Results

### 3.1 Basic characteristics

A total of 41 patients met the inclusion criteria (Group A *n* = 12; Group B *n* = 13; Group C *n* = 16). The median age was 9 (3–17) years old; 21 patients were male (51.2%); there were 29 patients with SAA (70.8%) and 12 patients with VSAA (29.2%); and 19 patients had a low bone marrow cellularity (46.3%) and 22 patients had an extremely low bone marrow cellularity (53.7%). The median duration from diagnosis to the use of ATG was 80 (18–407) days in Group A, the median duration from diagnosis to the initiation of EPAG was 40 (0–413) days, and the treatment duration of EPAG was 250 (66–863) days in Group A and Group B. In each of the three groups, 1 patient had a low number of PNH clones before treatment, and the tests of all patients were negative for chromosomal breakage, comet assay, and chromosomal aberration analysis. All patients underwent genetic testing to identify specific gene mutations that cause or contribute to bone marrow failure; however, no congenital bone marrow failure-associated gene mutations or clonal chromosomal changes were found in any of the patients. The differences in the natural killer (NK) cell count, CD19^+^ lymphocyte count, CD3^+^ CD4^+^ lymphocyte count and percentage, CD3^+^ CD8^+^ lymphocyte count and percentage, and CD4^+^/CD8^+^ ratio were statistically significant among the three groups of patients (*P* < 0.05). However, the differences in the remaining indicators were not significant (Supplementary Table 1).

### 3.2 Treatment response

**FIGURE 1:**
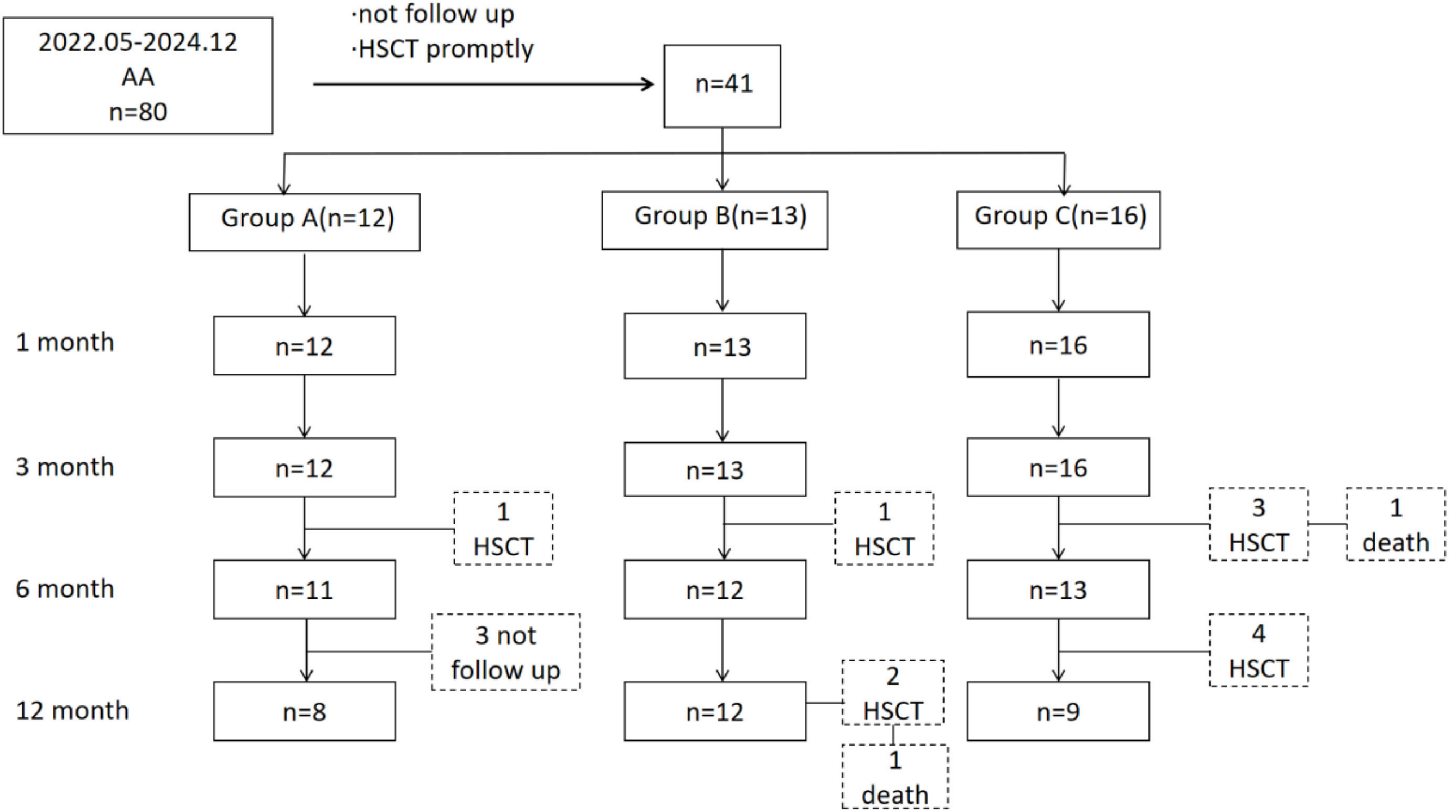
Patient-flow diagram.

By the follow-up cutoff time, 11 of 41 Patients underwent HSCT (1 patient in Group A, 3 patients in Group B, and 7 patients in Group C). 5 Patients underwent HSCT before 6 months of treatment (1 patient in Group A, 1 patient in Group B, and 3 patients in Group C), 4 patients in Group C underwent HSCT between 6 and 12 months of treatment, 2 patients in Group B underwent HSCT after more than 1 year of treatment. 3 patients in Group A did not reach the 12-month follow-up time (Figure 1). Except for patients who did not reach the follow-up time, patients who underwent HSCT before assessment as NR, there was no significant difference in the ORR among Groups A, B, and C at 1, 3, 6, and 12 months (*P* > 0.05) (Table 1).

**TABLE 1:** Comparison of efficacy among three groups[*n* (%)].

Time	Outcome	Group A	Group B	Group C	*P*-value
1 month (41)	PR	2 (16.7%)	6 (46.2%)	3 (18.8%)	0.162
	CR	0 (0.0%)	0 (0.0%)	0 (0.0%)	
	NR	10 (83.3%)	7 (53.8%)	13 (81.2%)	
	OR	2 (16.7%)	6 (46.2%)	3 (18.8%)	
3 month (41)	PR	5 (41.7%)	7 (53.8%)	4 (25.0%)	0.267
	CR	1 (8.3%)	0 (0.0%)	0 (0.0%)	
	NR	6 (50.0%)	6 (46.2%)	12 (50.0%)	
	OR	6 (50.0%)	7 (53.8%)	4 (25.0%)	
6 month (41)	PR	5 (41.7%)	6 (46.2%)	8 (50.0%)	0.177
	CR	4 (33.3%)	3 (23.1%)	0 (0.0%)	
	NR	3 (25.0%)	4 (25.0%)	8 (50.0%)	
	OR	9 (75.0%)	9 (69.2%)	8 (50.0%)	
12 month (38)	PR	3 (33.3%)	4 (30.8%)	8 (50.0%)	0.085
	CR	5 (55.6%)	5 (38.5%)	1 (6.3%)	
	NR	1 (11.1%)	4 (3.8%)	7 (4.8%)	
	OR	8 (88.9%)	9 (69.2%)	9 (56.3%)	

**FIGURE 2:**
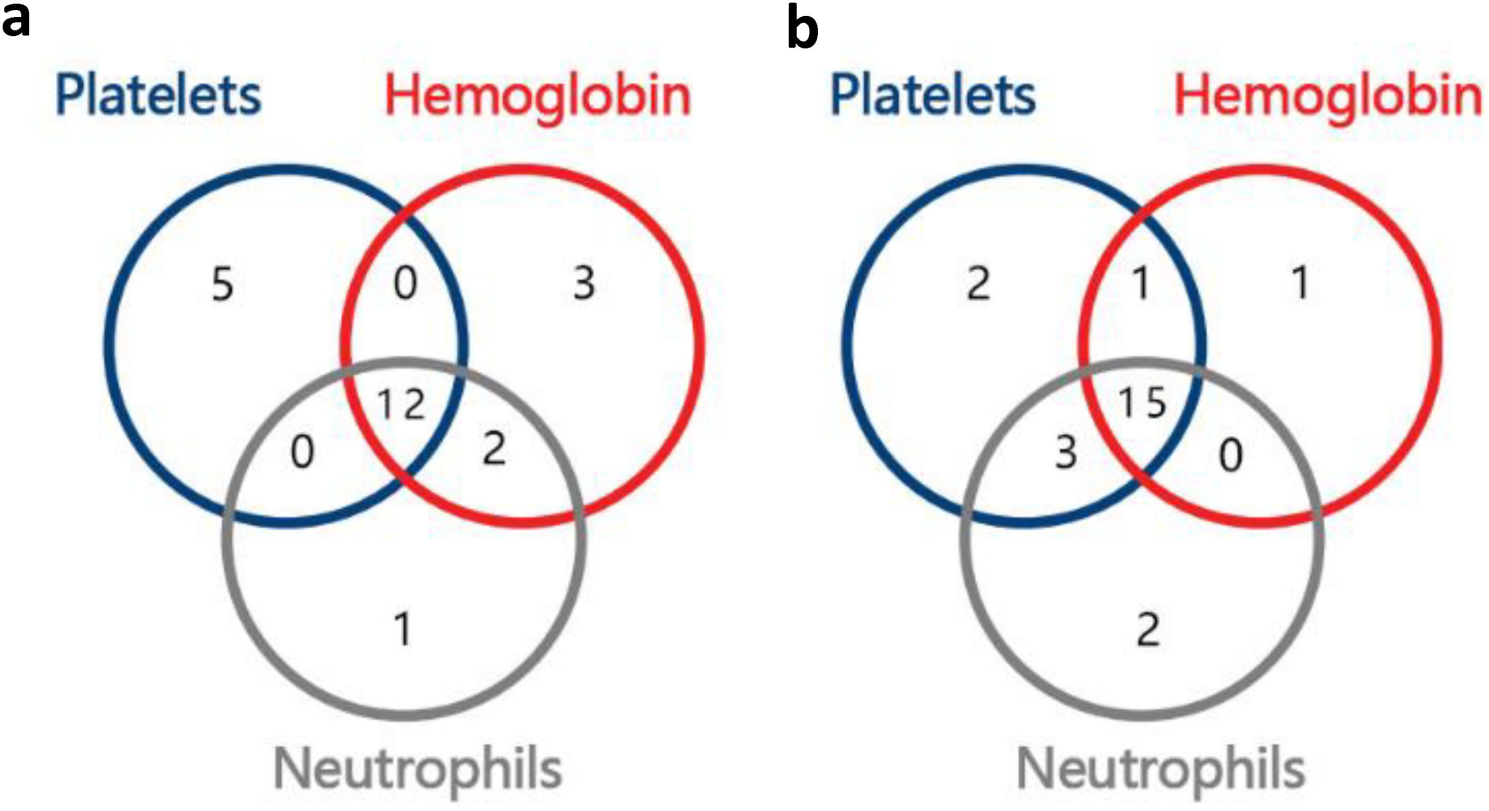
Number of responders with uni-, bi-, or tri-lineage response, **(a)**, 6th month; **(b)**, 12th month.

After 6 months of treatment, L% (lymphocyte percentage) in all three groups decreased, and the proportions of the bone marrow erythroid lineage increased; PLT count, Hb levels, and N% (neutrophil percentage) were increased in Group A; RET and the nucleated cell count increased in Group B; the WBC count and ANC increased in Group C; however, the differences were not statistically significant. There were significant differences in the NK cell count, CD19^+^%, CD3^+^CD4^+^% and count, CD3^+^CD8^+^% and count, CD4^+^/CD8^+^ ratio, and IgG levels (*P* < 0.05) among the three groups (Table 2). After 6 months of treatment, 33.3% (12/36) of the patients had a triple-lineage response, 22.2% (8/36) of the patients had a dual-lineage response, and 25% (9/36) of the patients had a single-lineage response. After 12 months of treatment, 48.4% (15/31) of the patients had a triple-lineage response, 9.7% (3/31) of the patients had a dual-lineage response, and 16.1% (5/31) of the patients had a single-lineage response (Figure 2).

**TABLE 2:** Comparison of index values at 6 months among groups (M ± SD).

Indicator	Group A	Group B	Group C	*P*-value
WBC (× 10^9^/L)	3.21 ± 1.16	3.81 ± 1.49	4.57 ± 2.69	0.246
PLT (× 10^9^/L)	90.81 ± 100.61	66.25 ± 58.61	46.46 ± 23.65	0.279
Hb (g/L)	102.18 ± 10.59	99.00 ± 22.38	91.76 ± 18.19	0.351
L (%)	39.97 ± 22.95	53.45 ± 13.75	53.96 ± 16.13	0.119
N (%)	52.38 ± 22.80	39.17 ± 13.23	39.30 ± 15.41	0.129
ANC (× 10^9^/L)	1.89 ± 1.23	1.59 ± 0.97	2.21 ± 1.89	0.570
RET (× 10^9^/L)	0.05 ± 0.03	0.06 ± 0.01	0.05 ± 0.03	0.780
Myeloid^△^ (%)	41.83 ± 18.64	40.33 ± 33.35	48.25 ± 24.00	0.910
Erythroid^△^ (%)	26.83 ± 15.41	52.16 ± 24.20	24.12 ± 14.97	0.170
Lymphocyte^△^ (%)	27.83 ± 29.82	16.00 ± 13.53	26.12 ± 21.24	0.784
CD34^+△^ (%)	0.06 ± 0.06	0.25 ± 0	0.15 ± 0.08	0.324
Nuclear counts (cell/μL)	3349.00 ± 377.59	36588.00 ± 0	13432.00 ± 14084.15	0.212
NK counts (cell/ μL)	143.28 ± 52.74	283.32 ± 42.34	237.18 ± 96.61	0.000***
NK (%)	12.85 ± 3.59	14.06 ± 0.12	10.88 ± 4.97	0.122
CD19^+^ counts (cell/μL)	160.86 ± 70.25	204.57 ± 40.20	194.50 ± 135.46	0.507
CD19^+^ (%)	18.58 ± 5.58	11.05 ± 3.11	8.35 ± 4.25	0.000***
CD3^+^CD4^+^ counts (cell/μL)	313.91 ± 139.17	758.41 ± 122.23	765.39 ± 232.79	0.000***
CD3^+^CD4^+^ (%)	30.90 ± 8.81	38.27 ± 5.76	35.31 ± 4.16	0.042*
CD3^+^CD8^+^ counts (cell/μL)	271.24 ± 106.15	537.18 ± 127.40	828.53 ± 316.31	0.000***
CD3^+^CD8^+^ (%)	27.80 ± 8.32	25.69 ± 2.35	37.11 ± 5.70	0.000***
CD4^+^/CD8^+^	1.28 ± 0.61	1.59 ± 0.38	0.98 ± 0.25	0.012*
IgG (g/L)	10.71 ± 1.99	14.00 ± 0.35	11.92 ± 3.97	0.018*

WBC, white blood cell; PLT, platelet; Hb, hemoglobin; L%, lymphocyte percentage; N%, neutrophil percentage; ANC, neutrophil; RET, reticulocyte. *P < 0.05, ***P < 0.001. ^△^In bone marrow.

### 3.3 Predictive factors

**FIGURE 3:**
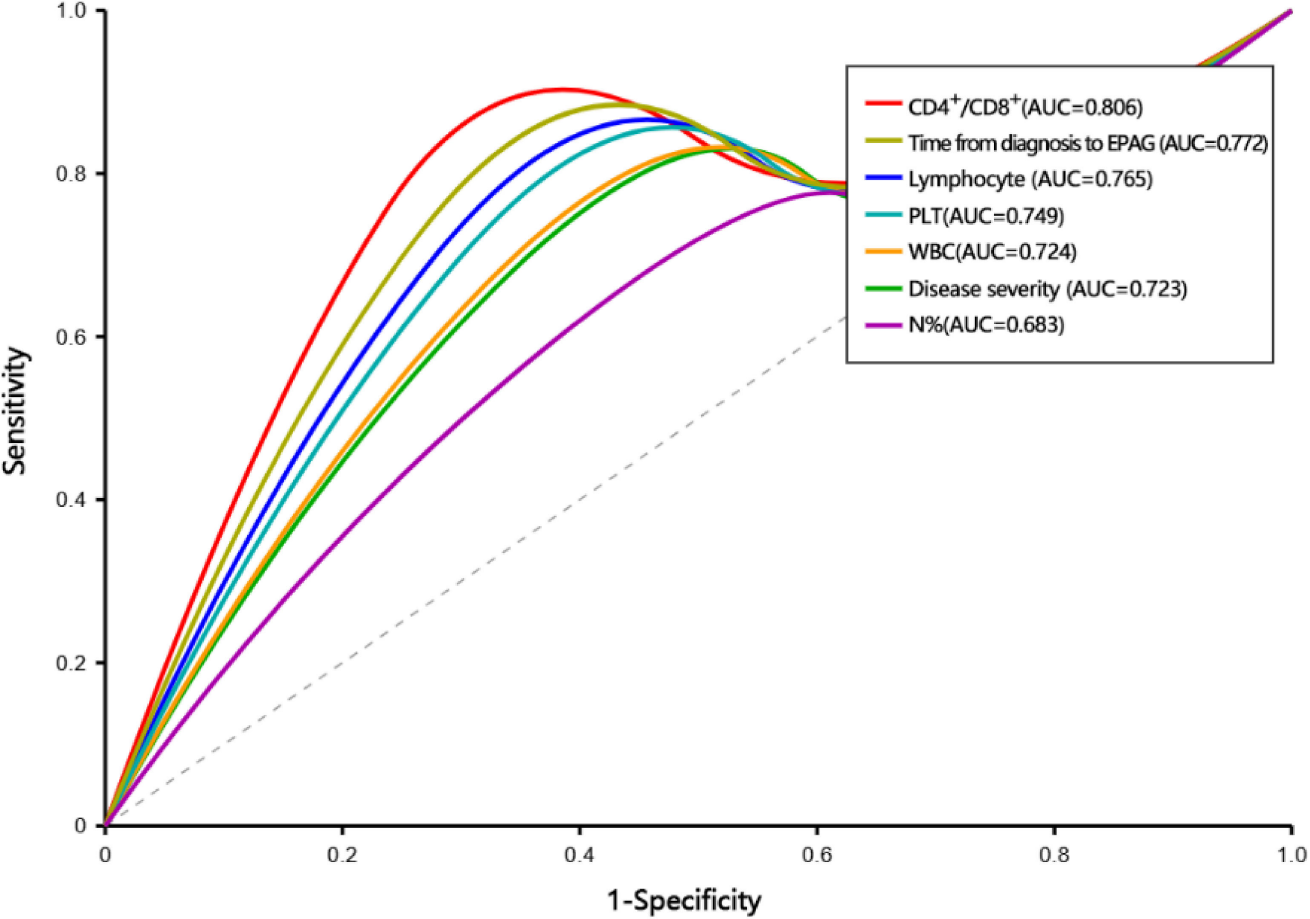
ROC curve for predictive factors.

One-way analysis of variance (ANOVA) revealed that the differences between the OR group and the NR group in the duration from diagnosis to receiving EPAG, WBC, PLT, N%, and CD3^+^CD4^+^% were significant (Supplementary Table 2). Each variable was included in the ROC curve. The factors that had a predictive value for OR were duration from diagnosis to receiving EPAG (AUC = 0.772, 95% CI 0.671–0.873, *P* = 0.013), severity of disease (AUC = 0.723, 95% CI 0.614–0.832, *P* = 0.027), the PLT count (AUC = 0.749, 95% CI 0.646–0.852, *P* = 0.018), the WBC count (AUC = 0.724, 95% CI 0.618–0.831, *P* = 0.035), N% (AUC = 0.683, 95% CI 0.573–0.793, *P* = 0.035), the lymphocyte count (AUC = 0.765, 95% CI 0.662-0.868, *P* = 0.012), and the CD4^+^/CD8^+^ ratio (AUC = 0.806, 95% CI 0.712–0.900, *P* = 0.004). Patients with a duration from diagnosis to receiving EPAG of ≤ 60 days, SAA, PLT ≥ 15 × 10^9^/L, WBC ≥ 2.0 × 10^9^/L, N% ≥ 40%, a lymphocyte count of ≥ 1.0 × 10^9^/L, and a CD4^+^/CD8^+^ ratio ≥ 1.5 were more likely to achieve a hematopoietic response (Figure 3).

### 3.4 Adverse reactions

All patients treated with ATG in Group A developed serum sickness, which manifested as fever, rash, and joint and muscle pain, with concomitant rise in IL-6, IL-8, and IL-10 levels. The median time of occurrence was the 11th day after treatment. In addition to the incidence of adverse reactions to ATG, the incidence of other adverse reactions in Group A at 6 months was 36.4% (4/11). The incidence of adverse reactions in Group B at 6 months was 58.3% (7/12). If the treatment time of the EPAG was less than 6 months, the end of the drug treatment was used as the observed outcome point. A total of 4 patients discontinued treatment before 6 months: 1 patient discontinued treatment due to muscle pain, 2 patients discontinued treatment due to poor efficacy, and 1 patient discontinued treatment due to relatively severe liver and kidney damage. The incidence of adverse reactions in Group C was 69.2% (9/13). Most patients had more than one adverse reaction. The incidence of hyperuricemia was the lowest in Group A and the highest in Group C, and the difference was significant (χ^2^ = 6.744, *P* = 0.034). The incidence of other adverse reactions, such as azotemia, increased transaminase levels, increased bilirubin levels, and total adverse reactions, was not significantly different among the three groups (Table 3).

**TABLE 3:** Incidence of adverse reactions among groups [*n* (%)].

Event	Group A (*n* = 11)	Group B (*n* = 12)	Group C (*n* = 13)	*P*-value
Azotemia	4 (36.4)	4 (33.3)	2 (15.4)	0.453
Hyperuricemia	1 (9.1)	1 (8.3)	6 (46.2)	0.034*
Elevated creatinine	0(0)	2 (16.7)	2 (15.4)	0.370
Elevated bilirubin	1 (9.1)	3 (25.0)	2 (15.4)	0.586
Elevated transaminases	0(0)	3 (25.0)	2 (15.4)	0.219
Gingival hyperplasia	0(0)	0(0)	1 (7.7)	0.403
Vomiting	0(0)	0(0)	1 (7.7)	0.403
Rate of occurrence	4 (36.4)	7 (58.3)	9 (69.2)	0.264

*P < 0.05.

### 3.5 Survival analysis and clonal evolution

**FIGURE 4:**
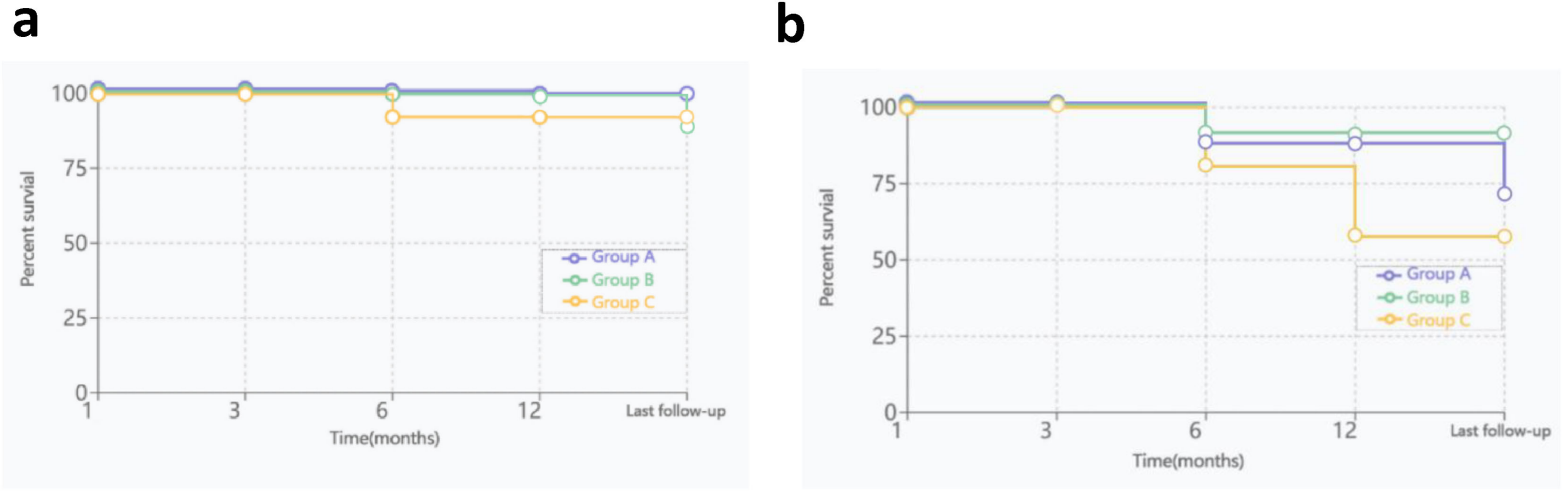
OS and EFS curves for three groups, **(a)**, OS curves; **(b)**, EFS curves.

By the cutoff time of follow-up, the OS rates of the three groups were 100, 92.3, and 93.8% (*P* = 0.649), respectively; the EFS rates of the three groups were 66.7, 69.2, and 56.3% (*P* = 0.130), respectively (Figure 4). 11 patients underwent HSCT, 2 patients died, and the rest survived. There was no significant difference in the OS rate between HSCT group and non-HSCT group (91.7%, 94.4%; *P* = 0.753). Only 1 patient in Group B had low-level paroxysmal nocturnal hemoglobinuria (PNH) clones according to flow cytometry, and this patient also had low-level clones at the beginning of the disease; the remaining patients did not undergo clonal evolution.

## 4 Discussion

Pathogenic factors induce antigenic alterations in the hematopoietic stem cells of AA patients, which subsequently activate T lymphocytes. This activation leads to significant changes in T-cell subset proportions, causing an abnormal immune response to kill HSPCs. The characteristic immunophenotypic changes include: CTL-mediated cytotoxicity; Th1/Th17 skew, reduced regulatory T cells (Tregs), and an increased CD4^+^/CD8^+^ ratio (11). EPAG can reduce the number of cytotoxic T cells, promote the release of transforming growth factor-β (TGF-β), increase the number of Tregs, and cause delayed macrophage activation and disordered dendritic cell maturation, which can be combined with the immunosuppressive effect of IST to reduce abnormal immune damage and promote the recovery of bone marrow hematopoietic function. In humoral immunity, regulatory B cells can return to normal levels in healthy people after 6 months of IST treatment, and the number of regulatory B cells is related to the severity of the disease (12). The results of this study revealed that at the level of T-cell-mediated cellular immunity, the CD3^+^CD4^+^ lymphocyte count and percentage, and the CD4^+^/CD8^+^ ratio increased the most, and the CD3^+^CD8^+^ lymphocyte count and percentage decreased the most in Group B at 6 months (*P* < 0.05). At the level of B-cell-mediated humoral immunity, the percentage of CD19^+^ lymphocytes in Group A increased after treatment, whereas the percentage of CD19^+^ lymphocytes in the remaining two groups decreased. Cellular immunity and humoral immunity both recovered to a certain extent in three Groups after treatment and were restored more significantly in the combined treatment group. Approximately 70% of lymphocytes are T lymphocytes, the CD4^+^/CD8^+^ ratio is indirectly significant in demonstrating the immune aspect of AA. Furthermore, the variation in Treg count was not assessed in this study. Future investigations should include such analyses, as alterations in the CD4^+^/CD8^+^ ratio may be related to the Treg count. The percentage of NK cells increased in all three groups after treatment, with the greatest increase in Group B (*P* < 0.05). This finding was consistent with the conclusions of previous studies showing that the percentages of NK cells and their subsets in the peripheral blood lymphocytes of newly diagnosed SAA patients were decreased and significantly increased after IST (13). The inhibitory protein in serum that inhibits erythroid colony formation from hematopoietic stem cells is mainly IgG, and its level is reduced after standard IST treatment (14). However, in our study, after treatment, the IgG levels in both Group A and Group B increased, whereas those in Group C decreased; however, those in all three groups were still within normal limits, indicating that although the addition of EPAG cannot effectively reduce the production of IgG, it does not enhance its inhibitory effects.

In this study, after 6 months of treatment for patients in the three groups, the patients with triple-lineage responses all had different degrees of increases in Hb, PLT and neutrophils. The increases in the Hb and PLT were the most significant in Group A, which was consistent with previous domestic and international studies (15). The depletion of T lymphocytes through complement-dependent cytolysis is the most important therapeutic mechanism of ATG (16). In this study, the lymphocyte percentages in the three groups decreased after treatment. Although there was no significant difference among the groups, the decrease was the greatest in Group A. After treatment, the proportions of bone-marrow erythroid lineage proportion increased in all three groups, with the increase in the BM nucleated cell count of Group B being the greatest. Compared with standard IST treatment, EPAG has a relatively strong effect on promoting PLT count, restoring immune function, and promoting the proliferation and differentiation of bone marrow hematopoietic stem cells. The initial effects of standard IST treatment manifest slowly for most patients; 50% of patients do not achieve significant results after receiving treatment for more than 3–6 months (17). EPAG combined with standard IST can significantly improve the early hematopoietic response rate of SAA patients. The 3-month hematopoietic response rate can reach 60–89%, and the CR rate can reach 30–44% (18). Except for the first month after the treatment, the ORR of patients reached more than 50% during the remaining follow-up time in this study, and the continuous use of EPAG effectively increased the rate of CR in PR patients. The ORR of patients across the three groups showed an increase, with the ORR in the combined treatment group demonstrating a notable rise compared to that of the monotherapy group. However, this difference did not achieve statistical significance. The small number of patients in the subgroup analysis, particularly in the IST group, likely reflects the limited efficacy of monotherapy, as a higher proportion of patients were either lost to follow-up or proceeded to underwent HSCT over the extended follow-up period.

In addition, the median time to achieve a hematopoietic response in the combined treatment group was shorter; one study reported that the median times to achieve a hematopoietic response with EPAG combined with IST treatment and IST alone were 105 and 184 days, respectively (8). In refractory AA patients, EPAG also showed a good hematopoietic response (19). However, not all studies have shown that EPAG has a good treatment effect. A retrospective study compared the treatment efficacy in treatment-naive SAA patients. The results revealed that EPAG did not improve the ORR at 6 months, and the response rate of young children (< 12 years) was lower than that of teenagers (≥ 12 years) (20). In this study, the median hematopoietic response times of CR patients in the three groups were 5, 7, and 13 months, in Group A, Group B, and Group C respectively, and the increase in the PLT count was earlier than that in the erythroid and granulocyte counts. EPAG significantly improved the remission rate of patients with RET count between 10 and 30 × 10^9^/L; the ORR increased from 60 to 91%. Regardless of whether IST was combined with EPAG, higher absolute lymphocyte counts were correlated with CR, especially in adolescents ≥ 10 years old, but the correlation between absolute lymphocyte counts and CR was the opposite in children < 10 years old (21). In prospective studies of treatment-naive SAA patients aged 3–82 years old, the earlier the medication time was and the longer the medication duration was, the higher the remission rate. However, owing to the short follow-up time, it is not suitable for all AA patients (22). This study also revealed that the stronger the residual hematopoietic function is, the sooner the IST and EPAG combination treatment can be initiated, which results in a higher CD4^+^/CD8^+^ ratio and more robust hematopoietic responses; however, the duration of treatment and RET count do not have high predictive value for treatment efficacy. Furthermore, research has found that the late initiation of treatment leading to poor efficacy may be related to the decrease in the number of remaining hematopoietic stem cells (19). hATG and rATG exhibit comparable ORR in SAA pediatric patients. However, the OS associated with hATG is significantly higher than that of rATG, which may be attributed to the more potent immunosuppressive effects of rATG leading to an increased risk of infections (23). Studies have shown that varying doses of rATG do not significantly influence OS, nor are they associated with elevated short-term adverse events or early mortality (24). Given that rATG was exclusively used in this study, a comparative analysis of the efficacy between different ATG types was not feasible.

EPAG usually leads to transient liver function damage, which manifests as increased indirect bilirubin levels, jaundice, and elevated aminotransferase levels (25), as well as skin rash and osteonecrosis of the femur (26). The incidence of elevated bilirubin in patients treated with EPAG combined with IST was 66.6% vs. 20.5% of those in the IST group (*P* < 0.05) (4), and the incidence of adverse reactions in patients with increased aminotransferase levels was 15–30%, which was significantly higher than that in patients treated with avatrombopag (27). Although the transient elevation of aminotransferase levels is not life-threatening, long-term monitoring and timely adjustment of the drug dose are needed. In this study, the incidence of elevated aminotransferase levels in Group B was 25%, and the difference in the incidence of adverse reactions among the three groups was not significant, which was consistent with the findings of previous studies.

Prior studies reported that although the OS rate of patients underwent HSCT was 1.769 times higher than that of patients receiving non-HSCT therapy (*P* > 0.05), EPAG combined with IST therapy was the treatment option that increased the OS rate the most among non-HSCT treatments (28). In this study, the number of underwent HSCT patients in the combined EPAG group was lower than that in the CsA alone group, with a statistically significant difference. This finding indirectly indicated that EPAG had a contribution to CsA alone treatment. The OS rates of both HSCT group and non-HSCT group were higher than 90% (*P* > 0.05), which was slightly higher than that reported in a previous study (29), which may be related to the short follow-up time in the present study. Groarke et al. (20) reported that the OS rate of the EPAG combined with IST treatment group was higher than that of the standard IST treatment group; the difference was not significant, but the combined treatment group had a greater tendency toward recurrence and a significantly reduced EFS rate. In this study, the OS rate and EFS rate of Group A were highest, there were not significantly different among three groups. At the last follow-up the OS rate was approximately 95.3%, which was consistent with that of a previous study (30). Zhang et al. (31) incorporated 16 studies involving a total of 2,148 patients. The analysis revealed that the response rate in the EPAG + IST group was significantly higher than that in the IST group at both 3 and 6 months. However, at 12 months, the difference between the two groups was not statistically significant. Compared with the IST group, the EPAG + IST group demonstrated superior OS rate, which aligns with the higher response and survival rates observed in this study. In addition, the OS rate of patients receiving standard IST treatment is strongly age dependent; studies have shown that the 5-year OS rate of patients < 40 years old is 90%, the 5-year OS rate of patients 40–60 years old is 80%, and the OS rate of patients over 60 years old is only 50% (32). All patients in this study were children, so age stratification was not performed. However, age is still a robust predictor of treatment response and survival.

Prior studies report that approximately 15% of AA patients undergo clonal evolution, including PNH and MDS/AML (33), from months to years after diagnosis (34). High-risk clonal evolution refers to the presence of chromosome 7 abnormalities, complex karyotypes, or obvious myeloid malignancies. Other isolated chromosomal abnormalities are considered low-risk clonal evolution (20). Compared with that of patients treated with HSCT, the EFS rate of patients treated with IST is significantly lower, with approximately 30% of patients experiencing recurrence and approximately 8–18% of patients exhibiting clonal evolution (15). The risk factors for clonal evolution in AA patients include age, no response after 6 months of IST treatment, a long course of disease (35), increased telomere attrition, and the presence of gene mutations that cause poor prognosis (36). EPAG functions by binding to the thrombopoietin receptor on megakaryocytes. Continuous receptor stimulation may promote the clonal evolution of AA into MDS/AML. The mutation proportion of myeloid cancer genes did not increase after the application of EPAG. The temporal relationship between clonal evolution and drug exposure indicated that EPAG may promote an increased probability of clonal evolution in patients with abnormal karyotypes (37). A very small number of patients develop myelofibrosis or secondary MDS, and the incidence rate is not significantly different from that of patients receiving IST alone (27); however, the time of recurrence and clonal evolution when EPAG is combined with IST treatment is earlier (38).

No significant clonal evolution was observed during the follow-up period in this study. Consistent with previous studies, the addition of EPAG did not lead to the development of myelodysplasia or hematopoietic malignancies (26), all the patients had normal bone marrow chromosomes at the last follow-up, and there was no abnormal MDS-related clone (25). The follow-up time of this study was short. The follow-up time needs to be extended in the future to determine whether the EPAG aggravates clonal evolution. In particular, patients with risk factors for clonal evolution should be followed up regularly even after achieving good long-term hematopoietic remission.

In conclusion, EPAG combined with IST for the treatment of children with AA showed a good hematopoietic response, and patients with a short time from diagnosis to receiving EPAG, SAA, and stronger residual hematopoietic function were more likely to achieve remission, had a high survival rate and EFS, and had no significant adverse reactions or cloning disorders. This treatment can overcome the limitation of conventional IST to a certain extent. However, the number of patients included in this retrospective study was limited, the number of subgroup analyses was small, and the follow-up time was short. Therefore, the number of samples and the follow-up time need to be increased in future studies. In combination with the precise treatment strategy of genomics, more personalized and effective treatment plans for children with AA can be developed.

## Data Availability

The original contributions presented in the study are included in the article/Supplementary material, further inquiries can be directed to the corresponding author.
