# Supplementary material for: Efficacy and influencing factors of immunosuppressive therapy combined with or without eltrombopag in children with severe aplastic anemia
Source: Front Med (Lausanne). 2025 Nov 21;12:1688771. doi: 10.3389/fmed.2025.1688771 (PMC12678098; doi:10.3389/fmed.2025.1688771)
Supplement: Supplementary file 1 [file Table_1.docx]

Supplementary Material

**Supplementary Table 1 Basic clinical characteristics of the patients**

| **Indicator** | | | | **Group A**  **(n=12)** | | | **Group B**  **(n=13)** | | **Group C**  **(n=16)** | | ***P-*value** |
| --- | --- | --- | --- | --- | --- | --- | --- | --- | --- | --- | --- |
| **Median age at diagnosis (range), years** | | | | **7 (3-17)** | | | **11 (5-16)** | | **9 (3-15)** | | **0.050** |
| **Gender, n. of patients (%)** | | | |  | | |  | |  | | **0.076** |
| **male** | | | | **3 (25.00)** | | | **9 (69.23)** | | **9 (56.25)** | |  |
| **female** | | | | **9 (75.00)** | | | **4 (30.77)** | | **7 (43.75)** | |  |
| **Severity of AA, n. of patients (%)** | | | |  | | |  | |  | | **0.392** |
| **SAA** | | | | **8 (66.67)** | | | **8 (61.54)** | | **13 (81.25)** | |  |
| **VSAA** | | | | **4 (33.33)** | | | **5 (38.46)** | | **3 (18.75)** | |  |
| **Time from diagnosis to EPAG (range), days** | | **84（29-413）** | | | | **40（0-407）** | | **NA** | | | **NA** |
| **Time from diagnosis to ATG (range), days** | | **80（18-407）** | | | | **NA** | | **NA** | | | **NA** |
| **WBC[×10^9^/L,M±SD]** | | **2.40±1.06** | | | | **3.21±1.57** | | **2.91±1.26** | | | **0.352** |
| **PLT[×10^9^/L,M±SD]** | **13.00±5.07** | | | | | **19.33±9.78** | | **15.30±14.75** | | | **0.378** |
| **Hb[g/L,M±SD]** | **80.63±17.37** | | | | | **88.75±16.03** | | **78.38±23.06** | | | **0.386** |
| **L[%,M±SD]** | | | | **65.69±24.08** | | | **58.32±15.39** | | **73.66±12.99** | | **0.113** |
| **N[%,M±SD]** | | | | **29.77±23.10** | | | **36.40±14.47** | | **21.69±10.68** | | **0.098** |
| **ANC[×10^9^/L,M±SD]** | | | | **0.53±0.35** | | | **0.86±0.40** | | **0.57±0.37** | | **0.056** |
| **RET[×10^9^/L,M±SD]** | | | | **0.02±0.02** | | | **0.01±0.01** | | **0.03±0.02** | | **0.085** |
| **EPO[mIU/mL,M±SD]** | | | | **182.38±138.15** | | | **304.20±228.06** | | **573.27±383.51** | | **0.153** |
| **TPO[pg/mL,M±SD]** | | | | **514.45±397.31** | | | **978.77±879.96** | | **741.72±552.79** | | **0.521** |
| **Myeloid^△^ [%,M±SD]** | | | | **16.75±14.30** | | | **27.90±16.54** | | **23.84±14.75** | | **0.344** |
| **Erythroid^△^ [%,M±SD]** | | | | **15.95±9.22** | | | **11.00±7.00** | | **16.80±13.83** | | **0.624** |
| **Lymphocyte^△^ [%,M±SD]** | | | | **63.48±19.10** | | | **57.30±21.99** | | **54.00±24.12** | | **0.599** |
| **CD34^+^counts^△^[cell/ul,M±SD]** | | | | | **9.76±18.03** | | **1.33±0** | | **11.86±15.78** | | **0.854** |
| **CD34^+△^[%,M±SD]** | | | | **0.07±0.11** | | | **0.03±0** | | **0.20±0.30** | | **0.632** |
| **Nuclear counts^△^**  **[cells/ul, M±SD]** | | | **8053.20±4348.75** | | | | **5316.00±0** | | **4117.00±3631.44** | | **0.344** |
| **NK counts[cells/ul,M±SD]** | | | | **233.66±100.70** | | | **126.99±41.62** | | **222.30±108.11** | | **0.015*** |
| **NK[%,M±SD]** | | | | **10.90±6.02** | | | **10.03±2.06** | | **8.21±3.96** | | **0.344** |
| **CD19^+^counts [cells/ul,M±SD]** | | | | **293.96±94.08** | | | **146.13±46.80** | | **438.14±176.99** | **0.000***** | |
| **CD19^+^[%,M±SD]** | | | | **12.75±4.00** | | | **12.64±3.48** | | **15.41±4.45** | | **0.201** |
| **CD3^+^CD4^+^counts [cells/ul,M±SD]** | | | | **1097.23±387.30** | | | **453.65±108.55** | | **1071.07±307.37** | **0.000***** | |
| **CD3^+^CD4^+^[%,M±SD]** | | | | **41.42±8.09** | | | **34.47±4.40** | | **38.67±4.71** | | **0.035*** |
| **CD3^+^CD8^+^counts [cells/ul,M±SD]** | | | | **655.38±198.61** | | | **524.18±176.64** | | **936.49±431.87** | | **0.008**** |
| **CD3^+^CD8^+^[%,M±SD]** | | | | **26.69±3.36** | | | **34.96±3.75** | | **32.20±7.69** | | **0.003**** |
| **CD4^+^/CD8^+^** | | | | **1.66±0.28** | | | **0.98±0.23** | | **1.35±0.59** | | **0.002**** |
| **IgG [g/L, M±SD]** | | | | **10.45±3.07** | | | **11.02±4.56** | | **12.81±2.69** | | **0.280** |

**n: number, WBC: white blood cell, PLT: platelet, Hb: hemoglobin, L%: lymphocyte percentage, N%: neutrophil percentage, ANC: neutrophil, RET: reticulocyte, EPO: erythropoietin, TPO: thrombopoietin,**

****P*<0.05, ***P*<0.01, ****P*<0.001.**

**^△^in bone marrow.**

**Supplementary Table 2 Univariate analysis of factors influencing efficacy after 6 months of treatment**

| **Indicator** | | **OR Group** | | **NR Group** | | ***P*-value** | |
| --- | --- | --- | --- | --- | --- | --- | --- |
| **Age[years,M±SD]** | | **9±3** | | **10±4** | | **0.471** | |
| **Gender[n(%)]** | |  | |  | |  | |
| **male** | | **11(42.3%)** | | **8(80.0%)** | | **0.098** | |
| **female** | | **15(57.7%)** | | **2(20.0%)** | |  | |
| **Diagnosis[n(%)]** | |  | |  | |  | |
| **SAA** | | **21(80.77%)** | | **6(60.0%)** | | **0.390** | |
| **VSAA** | | **5(19.23%)** | | **4(40.0%)** | |  | |
| **Time from diagnosis to EPAG[days,M±SD]** | | **52.30±28.40** | | **108.70±42.10** | | **0.013*** | |
| **Time from diagnosis to ATG[days,M±SD]** | | **92.22±122.68** | | **53.00±49.50** | | **0.678** | |
| **Duration of EPAG[days,M±SD]** | | **249.44±168.90** | | **275.40±331.24** | | **0.809** | |
| **WBC[×10^9^/L,M±SD]** | **2.68±1.21** | | **1.42±0.78** | | **0.021*** | |  |
| **PLT[×10^9^/L,M±SD]** | **18.70±9.30** | | **8.20±4.60** | | **0.018*** | |  |
| **Hb[g/L,M±SD]** | **82.40±18.70** | | **74.20±15.80** | | **0.321** | |  |
| **L[%,M±SD]** | **68.31±24.72** | | | **74.61±21.81** | | **0.412** | |
| **N[%,M±SD]** | **26.51±22.13** | | | **48.72±18.31** | | **0.035*** | |
| **ANC[×10^9^/L,M±SD]** | **0.68±0.57** | | | **0.82±0.61** | | **0.613** | |
| **RET[×10^9^/L,M±SD]** | **1.08±4.58** | | | **0.02±0.02** | | **0.549** | |
| **EPO[mIU/mL,M±SD]** | **354.81±267.42** | | | **412.64±293.12** | | **0.548** | |
| **TPO[pg/mL,M±SD]** | | **551.51±371.82** | | **411.60±517.46** | | **0.658** | |
| **Myeloid^△^[%,M±SD]** | | **20.84±11.94** | | **24.50±20.80** | | **0.550** | |
| **Erythroid^△^[%,M±SD]** | | **15.91±12.60** | | **15.44±11.20** | | **0.926** | |
| **Lymphocyte^△^[%,M±SD]** | | **58.45±19.94** | | **54.88±27.24** | | **0.694** | |
| **CD34^+^counts^△^**  **[cell/ul,M±SD]** | | **11.16±15.81** | | **1.03±0.52** | | **0.404** | |
| **CD34^+△^[%,M±SD]** | | **0.17±0.23** | | **0.04±0.01** | | **0.435** | |
| **Nuclear counts^△^**  **[cell/ul,M±SD]** | **6114.70±4379.27** | | | **3076.00±1238.85** | | **0.369** | |
| **NK counts [cells/ul,M±SD]** | **183.36±117.10** | | | **297.80±95.05** | | **0.085** | |
| **NK[%,M±SD]** | **9.13±3.87** | | | **12.63±9.51** | | **0.217** | |
| **CD19^+^counts**  **[cells/ul,M±SD]** | **327.04±169.99** | | | **412.13±221.87** | | **0.408** | |
| **CD19^+^[%,M±SD]** | **13.87±5.00** | | | **12.92±3.34** | | **0.693** | |
| **CD3^+^CD4^+^ counts [cells/ul,M±SD]** | **989.55±431.22** | | | **1040.28±377.97** | | **0.831** | |
| **CD3^+^CD4^+^[%,M±SD]** | **40.82±6.06** | | | **29.74±5.54** | | **0.003**** | |
| **CD3^+^CD8^+^ counts [cells/ul,M±SD]** | **707.31±329.19** | | | **1001.57±593.92** | | **0.183** | |
| **CD3^+^CD8^+^[%,M±SD]** | **29.93±6.08** | | | **32.45±11.13** | | **0.512** | |
| **CD4^+^/CD8^+^** | **1.46±0.50** | | | **1.15±0.61** | | **0.243** | |
| **IgG[g/L, M±SD]** | **11.82±3.85** | | | **10.19±4.67** | | **0.396** | |

**n: number, WBC: white blood cell, PLT: platelet, Hb: hemoglobin, L%: lymphocyte percentage, N%: neutrophil percentage, ANC: neutrophil, RET: reticulocyte, EPO: erythropoietin, TPO: thrombopoietin,**

****P*<0.05, ***P*<0.01.**

**^△^in bone marrow.**
